# Dead Zone Minimization Using Variable-Delay Element in CP-PLL for 5G Applications

**DOI:** 10.3390/mi14010081

**Published:** 2022-12-29

**Authors:** Dharani Buddha, Umakanta Nanda

**Affiliations:** School of Electronics Engineering, VIT-AP University, Amaravati 522237, Andhra Pradesh, India

**Keywords:** phase-frequency detector, phase noise, variable-delay element, lock time, lock range

## Abstract

The deadzone occurring in a phase-frequency detector (PFD) is a critical parameter that affects the performance of a phase-locked loop (PLL). Though a fixed-delay element reduces the deadzone, it creates an imbalance in the pulse-arrival time and among the up and down signals to the charge pump, which increases the phase noise in the output spectrum of the PLL. Therefore, in this work a new variable-delay element (VDE) is incorporated in the PFD to reduce the dead zone and consequently the phase noise of the PLL. The performance of the proposed PFD incorporated in PLL is analyzed using cadence virtuoso 90 nm CMOS technology, achieving a phase noise of −148.89 dBc/Hz at a frequency offset of 1 MHz, a lock time of 6.01 us, a power of 0.056 mW, and a dead zone of 110.5 ps, while operating at 3.5 GHz of frequency, making it suitable for 5G applications.

## 1. Introduction

Phase-locked loops (PLLs) are being focused on in research for their challenging aspects such as less dead zone, low phase noise, and less reference spur. The research in this field is looking for an additional wireless spectrum to offer higher capacity beyond 4G standards due to the need for faster mobile broadband connection. PLL is a widely used method for frequency synthesis and serves as the backbone for wireless transceiver systems. The implementation of the PFD, CP, VCO, loop filter, and frequency divider are the crucial elements defining in-band efficiency for an analogue-intensive PLL in terms of the trade-off among power and noise. The charge-pump phase-locked loop (CP-PLL) [1] consists of blocks such as the voltage-controlled oscillator (VCO), phase-frequency detector (PFD), charge pump (CP), loop filter, and frequency divider. The CP-PLL is chosen from among many types of the PLLs because it is very easy to integrate in micro-circuit devices.

Each block of the PLL governs a particular perspective of the signal, such as bandwidth consistency, spur reduction, phase noise, and wide lock range. The PFD is the second most important block of the PLL after VCO. In general, all parts of the PLL contribute to phase noise, with the VCO being the main source of noise. However, the noise generated by the PFD cannot be neglected. This noise from the PFD becomes prominent when the dead zone and reference spur goes beyond the limit.

In responding to the input reference $CK_{ref}$ and feedback signal $CK_{Out}$, the PFD produces two signals, up and down, according to the generic phase-locked loop. The noise generated by the PFD modulates the widths of the up pulses and down pulses, resulting in an arbitrary element in the output current of the charge pump. Three scenarios could be drawn from this. Where the CP produces no net output, the phase noise can modulate both the widths of outputs of the PFD in equal quantity. Only with respect to the down signal can the position of the up signal be modulated by phase noise. Mostly, the intriguing part is when the phase noise [2,3] modulates both the width of the up and down signals simultaneously. Consequently, the random difference among the widths of the up pulses and down pulses appears to increase the phase noise of the PFD.

Hence, one of the critical building blocks is the PFD as it is the block that is also responsible for measuring the phase difference between the input and reference signals. This phase difference [4] has a reasonable effect on the PLL’s overall performance, such as speed, locking ability, and noise performance. When the inputs vary in frequency, the phase difference changes every cycle by

(1) $$\Delta \varphi =2\pi \left[\frac{\left(T_{ref}-T_{out}\right)}{\max(T_{ref},T_{out})}\right]$$

Here, $T_{ref}$ and $T_{out}$ are the time periods of the input reference $CK_{ref}$ and feedback signal $CK_{out}$, respectively. As a result, an efficient PFD circuit should be developed [4,5,6] to effectively identify any phase difference and minimize as much risk as possible. The dead zone phenomenon is mostly responsible for an increased amount of phase noise in PFD. Whenever the positive-going edges of the two inputs to be matched are close to one another (or at around 360° of phase difference), the dead zone arises where the phase difference is quite minimal. If the PFD is in the dead zone, this could recognize phase errors and the PLL may get an erroneous condition and lock to the wrong phase. Therefore, the dead zone [7] must be successfully controlled.

As the noise [8,9,10] modulates the performance of the PFD significantly, in recent decades, designing an efficient PFD has become a challenging area for researchers, reducing phase noise by managing the dead zone. As reported by [2], the dead zone is frequently caused by the pre-charging time for the internal parasitic capacitances. Hence, they designed a PFD that diminishes the need for pre-charging. This method minimizes the dead zone to within the conceptual constraints required by process voltage temperature (PVT) differences. Such a PFD is consistent with the previously published high-speed PFD in [11], but it has been merged with a delay cell and the following two transistors. Retaining the frequency of operation at a nearly constant rate, the dead zone noted on this is 61 ps where, as in [6,12], the dead zone is reported as 156 and 221 ps, respectively. However, in [2] the phase-noise information and the lock time of the PLL require further research. When contrasted to its type-I counterpart, a classic tri-state phase-frequency detector [13] has the advantages of circuit robustness and a broad acquisition range. Nevertheless, the dead-zone concern of the tri-state PFD can cause a slowdown in the speed of the operation when driving the charge pump. Another work [14] described a dual-loop type-II PLL that used many power-hungry CML-type sub-divisions, including a frequency detector, divider-by-2 phase detector. However, dead-zone power consumption can further be improved. A calibrated PFD [4] technique is utilized to reduce the reference spur sustained without the dead zone. In its reset path, the suggested circuitry employs a PFD with a variable-delay element, with the delay length managed by feedback from the CP. However, the circuitry is complex, and the the phase noise, dead zone, power consumption, and lock time are not shown.

After going through the above previous work and understanding its limitations, this paper presents a phase-frequency detector with a variable-delay element in its reset path, reducing phase noise by controlling the dead zone while consuming low power. Here, a novel PFD is incorporated in the phase-locked loop, and the lock range, lock time, power consumption, and dead zone are observed.

## 2. Phase-Frequency Detector

The phase difference of both of the input signals from the frequency divider and the reference signal, when fed to the PFD, produces the outputs, which are denoted as up and down signals. The phase difference among two input signals can be used to calibrate the width of these up and down signals. Based on these signals, the VCO alters the frequency of the output. The VCO enhances its output frequency whenever the reference signal precedes the feedback input signal. Consequently, the VCO lessens its output frequency whenever the reference signal stagnates behind the feedback input signal. The PLL becomes locked to the invalid phase or the frequency if the PFD is unable to identify the accurate phase or the frequency difference. Both up pulses and down pulses of one of the inputs of the PFD at the rising edge are low; then, the correlated output up or down goes high, and this is repeated till the input becomes low and the second input becomes high. Whenever two outputs become high, device resets. The PFD will be set or reset by these signals. When the PLL is about to lock, there is a minor phase difference in the two inputs.

If a rising edge is observed in either of the signals, the up or down signals will take a finite amount of time to propagate and switch on the CP. In this time frame, if the rising edge of the another input is found, at that moment the output becomes high again. Whenever two PFDs’ outputs become high, a reset signal is produced. However, the amount of time for which both the up and down signal will be high is known as the dead zone, and at this duration the PFD will not be able to identify the phase differences. The output frequency of the PLL tends to vary, causing an increase in the overall phase noise. Regarding the standard PLL with a frequency divider in its feedback in [15], the phase-noise spectral density to the divider ratio gain, *N*, is

(2) $$L\left(f\right)=8\pi^{2}f_{CK_{ref}}\Delta t^{2}N^{2}\frac{{\mathrm{rad}}^{2}}{\mathrm{Hz}}$$

where

$N=f_{VCO_{out}}/f_{CK_{ref}}$,
$f_{VCO_{out}}$ is the output frequency of the VCO,
$f_{CK_{ref}}$ is the operating frequency of the PFD,
Δt is known as blind zone and defined as the phase difference between two inputs of the PFD,
PFD’s gain attenuation factor [6] is
  (3) $$\alpha =1-2\left(\Delta t-T_{D}\right)-T_{D}^{2}$$
  (4) $$\;\mathrm{Thus},\;\Delta t=\frac{1-\alpha -T_{D}^{2}+2T_{D}}{2}$$
  where $T_{D}$ is delay of the delay element.
  Now, by solving Equations (2) and (4)
  (5) $$L\left(f\right)=8\pi^{2}f_{ref}{\left(8\pi \frac{1-\alpha -T_{D}^{2}+2T_{D}}{2}N\right)}^{2}\frac{{\mathrm{rad}}^{2}}{\mathrm{Hz}}$$

From Equation (3), it can clearly be observed that the phase noise is correlated to the delay width. However, the practically dead zone cannot be made zero.

**Figure 1:**
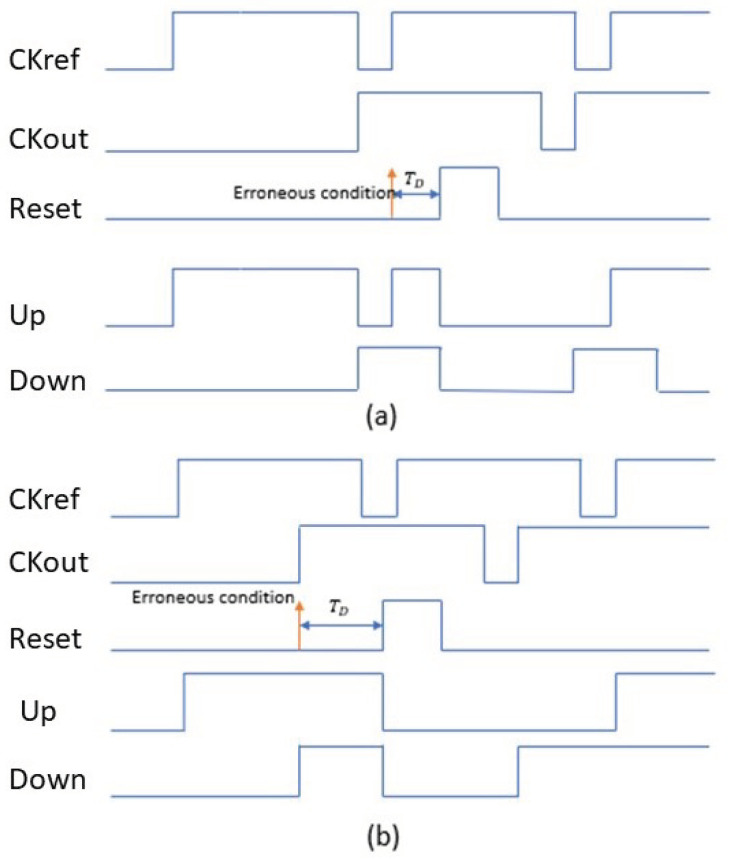
(**a**) Error caused by the RESET’s delay. (**b**) Error caused when RESET was delayed.

Researchers integrated a delay element block with a delay of $T_{D}$ in the PFD’s reset path. However, inside the above delay $T_{D}$, as seen in Figure 1a, the very next rising edge of the reference signal $CK_{ref}$ has no impact on the up signal, resulting in incorrect behavior due to the up signal already being turned on. As shown in Figure 1b, any rising edge of the $CK_{ref}$ identified during the high period of reset has no impact on the up signal. Due to the fact that the up signal’s rising edge was absent in both situations, the down signal resulted in a negative output, enhancing the difference in $CK_{ref}$ and feedback signal $CK_{out}$ in the form of phase and frequency for phase differences greater than 2π−2δ. Where, δ=2π$T_{D}$/$T_{CK_{ref}}$, the data about the phase or frequency difference immensely limits the effectiveness of the PFD and PLL.

**Figure 2:**
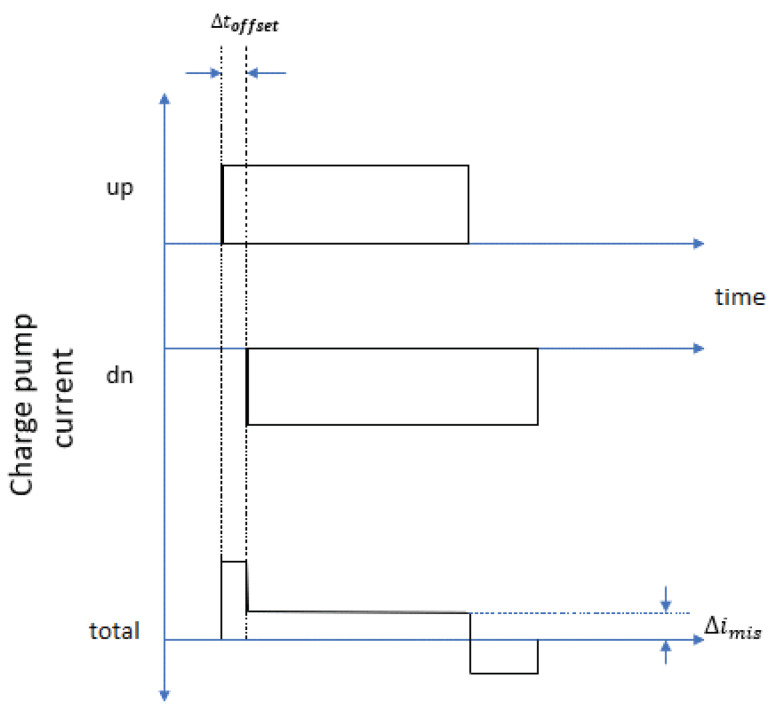
Impact of mismatches in charge-pump currents and pulse-arrival time.

The PFD cannot identify the phase difference, which is less than $\phi_{dz}$. Within this range, the phase at the PLL output tends to vary freely, leading to increased phase noise. The easiest solution to the dead-zone issue is by placing a fixed-delay element in the reset path of the PFD. This forces up pulses and down pulses to be active at the same time for a fixed period in every cycle whenever the PLL is locked. The disadvantage of the above technique is an imbalance in pulse-arrival times (as shown in Figure 2), and the up and down currents in the CP cause a periodic disruption of the control voltage of the VCO, resulting in reference spurs in the PLL output spectrum. Thus, the variable-delay element resolves this drawback since there is no fixed delay period and noW calls for a novel PFD.

## 3. Proposed PFD with Variable-Delay Element

**Figure 3:**
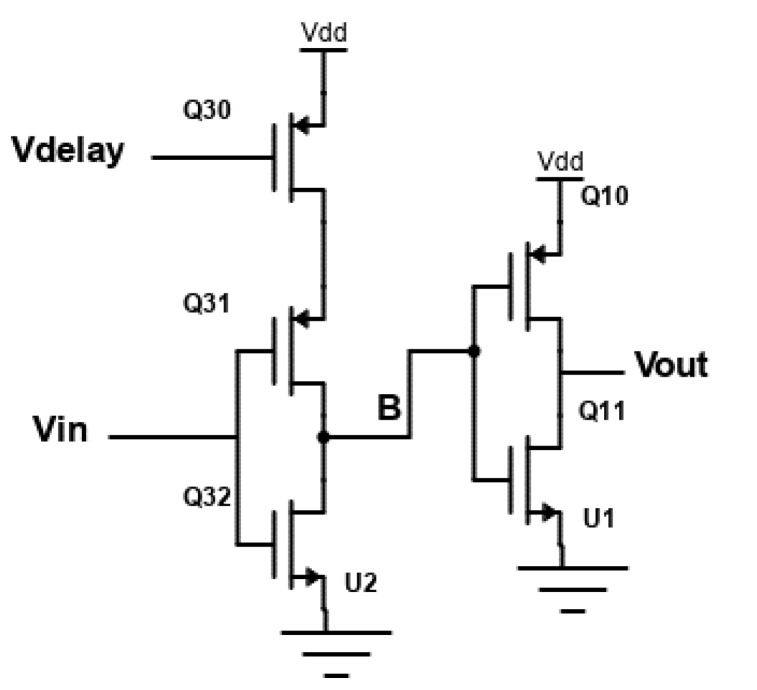
Schematic of proposed variable-delay element.

**Figure 4:**
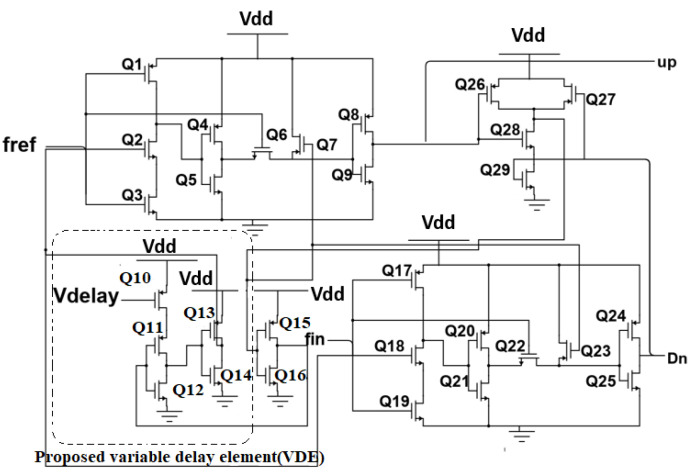
Circuit diagram of the PFD with proposed variable-delay element.

The input signals for various PLL applications differ. A fixed-delay element would not function efficiently to lessen the dead zone with these differences in input signals. If the fixed delay is greater than the required delay, the reference spurs will be enhanced. On the other hand, if the delay is below the desired value, the dead-zone issue will reoccur. To address this, a variable-delay element (Figure 3) is integrated in the reset path, ensuring that the overall delay is appropriate while accomplishing the simultaneous need of less phase noise and a minimum dead zone while making the PFD adaptable to PLLs operating at different frequencies. The proposed PFD has a variable-delay element (Q10–Q14) depicted in Figure 4, placed in the reset path of the PFD, which reduces the phase noise and the dead zone. The simulation and analysis are performed in a 90 nm CMOS process with a supply voltage of 1 V.

### Variable-Delay Element

The proposed variable-delay element is shown in Figure 3, which consists of two inverters, i.e., a current-starved inverter and standard inverter. The term “current-starved” in this perspective means that the current flowing through the circuit is really constrained. A “standard” inverter is connected to the supply rails and ground directly. It can theoretically draw as much current as it wants. When the PFD resets, it creates a dead zone on the down transition. Thus, the design confines the current flow only in this transition.

If the control voltage is increased, it slows down the transition by increasing the resistance included by the node “B” all through charging. Another inverter is not a current-starved inverter; thus, the output of the variable-delay element has usual rise and fall times.

However, predicting the average current, which comparable to the saturation current of the MOSFETs in second inverting stage, yields a fitting expression. From the channel-length modulation coefficient,

(6) $$\begin{array}{cc} I_{av}= & \frac{k_{p}}{2}{\left(V_{GS}-\left|V_{T_{P}}\right|\right)}^{2}\left(1+\lambda V_{DS}\right)\\ = & \frac{k_{p}}{2}{\left(V_{lD}-\left|V_{T_{P}}\right|\right)}^{2}\left(1+\lambda V_{DS}\right)|\\ \; & \approx \frac{k_{p}}{2}{\left(V_{DD}\right)}^{2}\left(1+\lambda V_{DS}\right) \end{array}$$

where λ is the channel-length modulation coefficient.

According to Equation (6), the second stage propagation delay can be conveyed as

(7) $$t_{p^{2}}=\frac{1}{2}\left(t_{pLH}+t_{pHL}\right)=\frac{c_{L}}{2V_{DD}\left(1+\lambda V_{DS}\right)}\left(\frac{1}{k_{p}}+\frac{1}{k_{n}}\right)$$

in which $t_{pLH}$ and $t_{HL}$ seem to be the propagation delays for low to high output transitions and high to low output switching, respectively. The equation for $t_{p^{2}}$ undergoes switching from $V_{DD}$ to ground or conversely.

In which,

$$k_{n}=\mu_{n}C_{OX}{\left(W/L\right)}_{n}$$

$$k_{p}=\mu_{p}C_{OX}{\left(W/L\right)}_{p}$$

The propagation delay of the delay element is,

(8) $$t_{p^{1}}=\frac{1}{2}\left(t_{pLH}+t_{pHL}\right)=\frac{c_{L}}{2V_{DD}\left(1+\lambda V_{DS}\right)}\left(\frac{1}{k_{p}}+\frac{1}{k_{n}}\right)$$

The total delay of the delay element $t_{p,total}=t_{p^{1}}+t_{p^{2}}$

Moreover, if the fixed delay is greater than required due to variations in input signals for various PLL applications, the reference spurs will be enhanced. From the other end of the spectrum, unless the delay is quite limited to keep the complete PFD delay positive, a modification in operating conditions will alter the overall PFD delay to a negative value, reintroducing the dead-zone issue. To keep away from this issue, a PFD with a VDE in its reset path is used, ensuring that complete delay is positive with fewer reference spurs and the least dead zone of the PFD’s output signals. The CP circuit converts output digital signals fed from the PFD to analog voltage, which is referred to as the control voltage of the VCO. The VCO produces a clock, and the frequency of the clock is managed by this control voltage $V_{cont}$, which also gets tuned by the loop filter. The loop filter block eliminates the high-frequency elements from the signal produced by the CP. By dividing the frequency of output of the VCO by a particular division, phase noise is limited. The frequency divider of ratio-16 is used to divide the output frequency in this design.

## 4. Results and Discussion

Further analysis and discussions are made by simulating proposed PFD, CP-PLL [1] in 90 nm CMOS technology in Cadence virtuoso tools with a supply voltage of 1 V. Various PLLs are simulated for the comparison of performance, which are demonstrated in this section.

**Figure 5:**
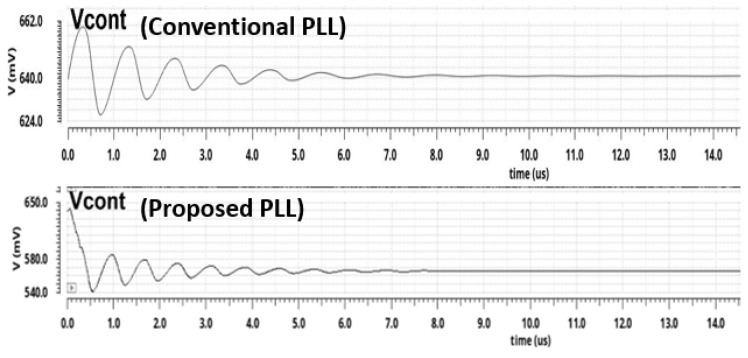
The control voltages of conventional and proposed PLLs.

The transient analysis of conventional PLL and novel CP-PLL are simulated, and their control voltages are presented in Figure 5. By varying the delay voltage, transient analysis is carried out. As there are no passive elements such as the R and C components, the CP-PLL consumes less area. The calculations and comparisons of the power of innovative CP-PLL and standard PLL are made. The power consumption of conventional PLL is 0.058 mW, whereas for novel CP-PLL it is 0.056 mW. As per the results shown here in Table 1, the proposed CP-PLL has slightly lower power than the standard PLL. The dead zones of the conventional PLL and the novel PLL observed here are 113.03 ps and 110.5 ps, respectively, and are shown in Table 1.

**Figure 6:**
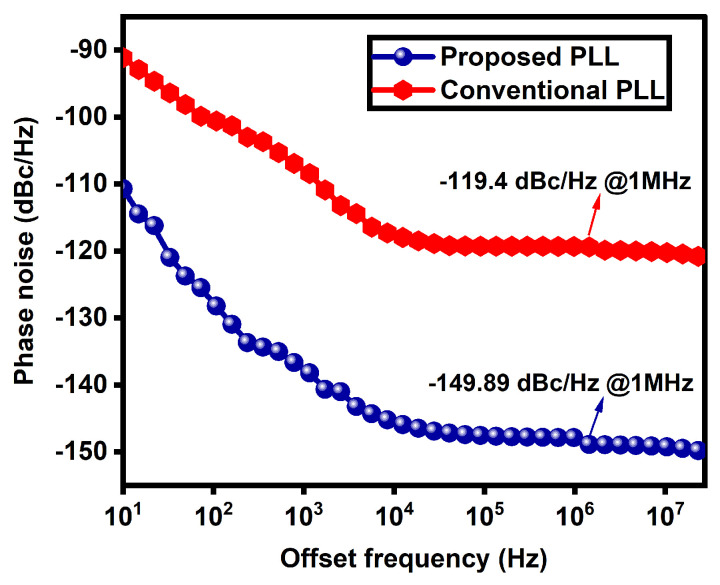
Comparison of the phase noise plots of novel PLL and conventional PLL.

The phase-noise plot comparison of conventional PLL and novel PLL is shown in Figure 6. The phase noise of novel PLL is −148.89 dBc/Hz at 1 MHz offset, and that of the conventional PLL is −119.4 dBc/Hz at 1 MHz offset, shown in Table 1. For the simulation of novel PLL, the delay voltage is 0.5 V. If the delay voltage is less than this, the PLL will not lock. From the results compared in Table 1, it can be observed that the novel PLL has less phase noise than its counterpart due to the variable-delay element. The novel CP-PLL also has a wide lock range of 3.12–14.01 GHz, a lower dead zone of 110.5 ps, and a frequency of 3.5 GHz.

### Corner Analysis

The proposed CP-PLL outperforms with regard to power, phase noise, dead zone, lock range, and lock time. Table 2 shows the results of the proposed CP-PLL and is further analyzed with temperature variations ranging from 27 degrees to −27 degrees across different corners (NN, FF, FS, SF, SS @27 °C, 0 °C, and −27 °C). From the above shown results, the phase noise is comparatively identical in all five corners; however, the FS is better and the SS is worse. With a decrease in temperature, the phase noise increases.

**Figure 7:**
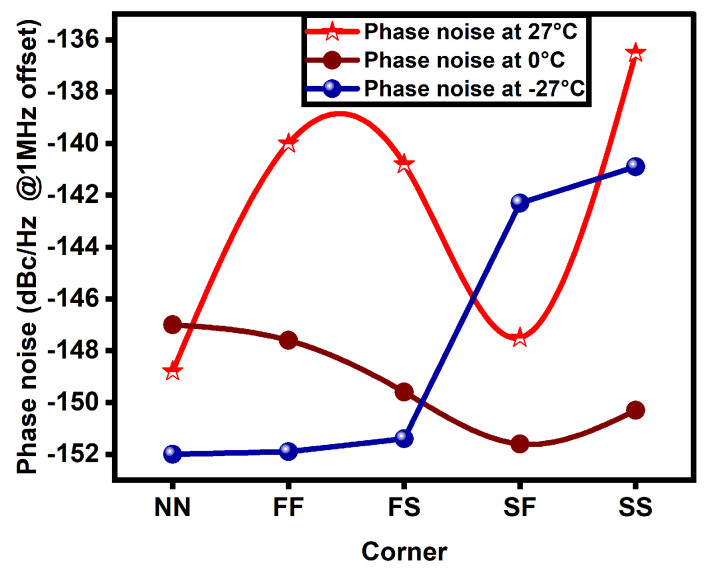
Phase noise comparison plot of proposed PLL among various corners at different temperatures.

**Figure 8:**
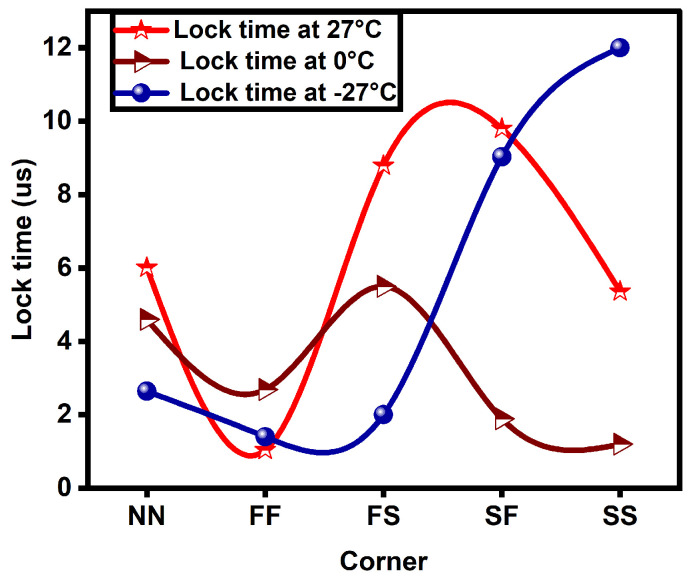
Lock time comparison plot of proposed PLL among various corners at different temperatures.

**Figure 9:**
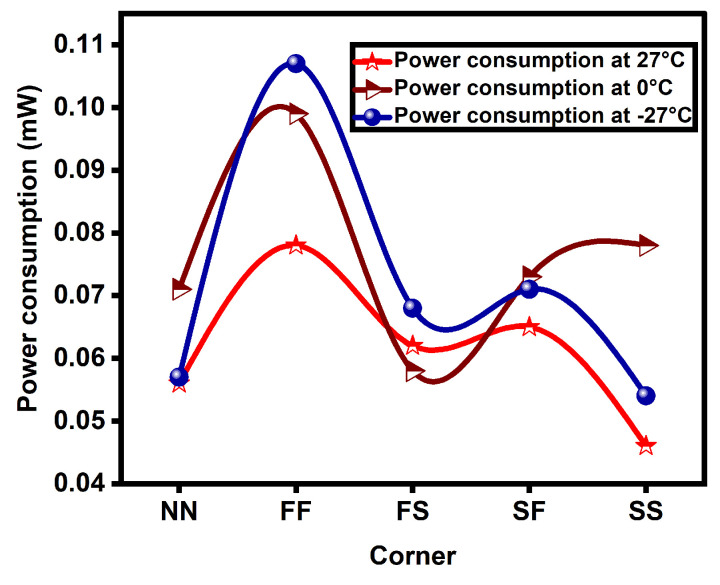
Power consumption comparison plot of proposed PLL among various corners at different temperatures.

From the results, it is also observed that power varies from one corner to the other. The power consumption of the novel PLL is high in FF and low in the SS corner. The novel CP-PLL is fast locking in the FF corner and slow in the SF corner. The phase noise, lock time, and power consumption plots are depicted in Figure 7, Figure 8 and Figure 9, respectively, and are compared in different corners such as NN, FF, FS, SF, and SS at various temperatures such as −27 °C, 0 °C, and 27 °C.

Table 3 compares the summary of the proposed CP-PLL to the recent works. As can be seen, previous research achieved a high oscillation frequency at the expense of increased power usage, a wide lock range, and increased phase noise, with the high dead zone rendering it unfit for the high-speed circuits.

## 5. Conclusions

In this work, the PLL is proposed with a variable-delay element in its reset path of the PFD. The above approach minimizes the dead zone, leading to improved phase-noise efficiency. This design’s lock in time and lock range are also noted. The lock range is found to be less than that of the traditional PLL, and the lock time of the PLLs is significantly shorter than that of the PLL without any delay. Compared to traditional design, this unique PLL saves power up to 4.92%, phase noise up to 24.6%, dead zone up to 2.23%, and lock time up to 12.8%. The novel CP-PLL is analyzed with previous works and is shown here. The proposed PLL is also compared in different corners with various temperatures, beginning with −27 °C to 27 °C. The novel CP-PLL has a phase noise of −148.89 dBc/Hz at 1MHz, power consumption of 0.056 mW, a dead zone of 110 ps, a lock time of 6.01 us, a lock range of 3.12–14.01 GHz, and a frequency of 3.5 GHz, at a supply voltage of 1 V. These characteristics were compared, and the results showed that the novel circuit can function more effectively while reducing the lock range, power, phase noise, and dead zone, and by increasing the lock time to acceptable levels.

## Figures and Tables

**Table 1:** Comparision of traditional PLL and novel CP-PLL parameters.

Parameters	Conventional PLL	Novel PLL
Tech (nm)	90	90
Freq (GHz)	3.5	3.5
Supply voltage (V)	1	1
Phase noise @1 MHz (dBc/Hz)	−119.4	−148.89
Dead zone (ps)	113.03	110.5
Power (mW)	0.058	0.056
lock time (us)	6.9	6.01
lock range (GHz)	3.1–8.2	3.12–14.01

**Table 2:** An overview of the new PLL’s performance at various process corners and temperatures.

Corner	Phase Noise (dBc/Hz at 1 MHz Offset)			Lock Time (µs)			Power Consumption (mW)		
	27 °C	0 °C	−27 °C	27 °C	0 °C	−27 °C	27 °C	0 °C	−27 °C
NN	−148.8	−147	-	6.01	4.6	2.64	0.056	0.071	0.057
FF	−140	−147.6	−151.9	1.04	2.69	1.4	0.078	0.099	0.107
FS	−140.8	−149.6	−151.4	8.79	5.5	2	0.062	0.058	0.068
SF	−147.5	−151.6	−142.3	9.8	1.89	9.03	0.065	0.073	0.071
SS	−136.5	-	-	5.36	1.2	12	0.046	0.078	0.054

**Table 3:** Overview of novel CP-PLL performance.

References	[16]	[17]	[18]	[19]	[20]	[21]	[22]	[23]	[24]	[25]	This Work
Tech (nm)	130	90	90	90	130 nm	90	90	180	28 nm	180 nm	90
PFD-type	PFD	VVDE-PFD	VDE-PFD	VVDE-PFD	Multi state- PFD	Latch based PFD	VDE-PFD	No reset path PFD	edge-triggered PFD	voltage-mode PFD	VDE-PFD
Freq (GHz)	1.5	2.5	1	-	1.5	1	1	3.4	3	3	3.5
Supply vol (V)	1.2	1.8	1.8	-	1.2	1.8	1.8	1.8	0.9	1.8	1
Power (mW) @ 1 MHz	2 @ 1 GHz	1 @ 500 MHz	2.31	1.74	25 @ 1 GHz	1.43	1.73	0.055	3	-	0.056
Pnoise (dBc/Hz)	−96.2	−110.5	−109.5	−110.5	−96.2	−90.7	−109.5	−147.86	−111	−107	−148.8
Dead zone (ps)	-	36	65	36	-	-	-	-	-	-	110.5
Lock time (µs)	2	0.05	0.09	0.06	2	0.22	0.05	-	-	10	6.01
Lock range (GHz)	-	-	0.55–2.5	0.8–2.5	-	0.5–1.9	0.7–2.1	-	-	-	3.12–14.01

## Data Availability

The authors confirm that the data supporting the findings of this study are available within the article, or below mentioned references.
